# Integrated targeted serum metabolomic profile and its association with gender, age, disease severity, and pattern identification in acne

**DOI:** 10.1371/journal.pone.0228074

**Published:** 2020-01-17

**Authors:** Min Hee Kim, In Jin Ha, Eunok Kim, Kyuseok Kim

**Affiliations:** 1 Department of Ophthalmology & Otolaryngology & Dermatology, College of Korean Medicine, Kyung Hee University, Seoul, Republic of Korea; 2 Korean Medicine Clinical Trial Center, Kyung Hee University Korean Medicine Hospital, Seoul, Republic of Korea; 3 College of Korean Medicine, Kyung Hee University, Seoul, Republic of Korea; Katholieke Universiteit Leuven Rega Institute for Medical Research, BELGIUM

## Abstract

**Background:**

Westernized diet and nutritional metabolism are important in acne pathogenesis, especially in adult patients. However, clinical and basic data are lacking. Pattern identification (PI) is a tool that results in a diagnostic conclusion based on a cluster of concurrent symptoms and signs in traditional medicine. Acne can be classified by PI. However, whether the metabolomic profile differs according to the PI of acne is unknown. Metabolomic data would help clarify the pathogenesis of acne.

**Methods:**

We conducted a cross-sectional study involving 40 healthy controls and 60 subjects with acne. We evaluated androgens, serum lipids, essential amino acids, nonessential amino acids, other amino acids, and pro-inflammatory cytokines of all subjects and compared the metabolomic profiles between acne subjects and healthy controls, and in subgroups according to gender, age, severity, and PI.

**Results:**

Dehydroepiandrosterone sulfate and serum fatty acids were significantly higher in female subjects, adolescents, and those with disharmony of the thoroughfare and conception vessels. The total essential and nonessential amino acids were significantly lower in the overall, female, adult, severe, and phlegm-stasis group. The latter group exhibited elevated serum levels of interleukin-1β and -6.

**Conclusions:**

This is the first study to investigate serum lipids, amino acids, and cytokines in subjects with acne. We analyzed the differences between metabolomic profiles to determine the diagnostic value of PI. Prospective studies with more patients are needed to confirm the characteristics of each PI and lipidomic data will enrich knowledge concerning lipid mechanism.

## Introduction

Acne vulgaris is a common chronic inflammatory dermatosis that affects 85% of adolescents and young adults worldwide [1–3]. Increased risk of acne has been described in developed countries compared with developing countries [4], which is closely related with the adoption of a more westernized diet. A recent study reported that acne prevalence in adults is increasing, especially in female subjects [5]. Adult acne or adult female acne is considered as a specific acne subtype distinct from adolescent acne [6]. Diet and nutritional metabolism play key roles in acne pathogenesis, especially in adult patients. However, clinical and basic research data are lacking.

In addition to diet, hormonal abnormalities [7], stress and modern life style [8], smoking [9], environmental factors [10, 11], and genetic predispositions [12, 13] may contribute to acne. The influence of these systemic factors on acne has recently changed the perception of acne as both a site-specific and systemic disease [14]. From this perspective, the development of a tool that classifies acne according to systemic symptoms is needed for the individualized and targeted therapy of acne. Pattern identification (PI) is a tool that results in a diagnostic conclusion based on a cluster of concurrent symptoms and signs in traditional medicine that is based on the themes of holism and individualization [15]. Although standardized diagnostic criteria have not been described in the classic literature, advancements in systems biology and computing techniques have led to progress in the standardization and modernization of PI [16, 17]. A standardization study revealed that acne also can be classified by PI [18]. However, the PI metabolomic profile indicating the difference of pro-inflammatory cytokines in acne is unknown.

Metabolomic analysis would provide more knowledge of the pathogenesis of acne. Thus, the aims of this study were to compare the metabolomic profiles with pro-inflammatory cytokines between acne subjects and healthy controls, and to compare the differences in these metabolomic profiles with pro-inflammatory cytokines in subgroups according to gender, age, severity, and PI.

## Materials and methods

### Study design

This cross-sectional study was conducted at Kyung Hee University Korean Medicine Hospital (Clinical Research Information Service, Republic of Korea: KCT0002212) [19]. Healthy volunteers and subjects with acne were enrolled at a screening visit and outcomes were measured after one week (Table 1). This study was approved by the institutional review board of Kyung Hee University Korean Medicine Hospital (KOMCIRB-161118-HR-062). Written informed consent was obtained by the investigator from all participants prior to enrollment.

**Table 1:** Study schedule.

Visit (week)	Screening (week 0)	Visit (week 0 to 1)
Informed consent	●	
Inclusion/Exclusion	●	
Demographics	●	
Physical examination	●	
Medical history	●	
Questionnaire^a^	●	
Korean Acne Grade Scale	●	
Skin assessment^b^		●
Blood collection^c^		●

^a^pattern identification inventory, smoking, sleep, stress response inventory, and dietary habits

^**b**^skin temperature, skin stiffness (cutaneous resonance running time), and sebum levels

^**c**^androgens, serum lipids, amino acids, and cytokines

### Sample size

This study was not intended to validate the effects of certain interventions on acne, but rather to compare metabolomic profiles with pro-inflammatory cytokines of acne subjects with those of healthy controls. Moreover, in the metabolomic study design, a sample size of three to five subjects per group may provide useful preliminary data [20]. Therefore, in this exploratory study, the number of subjects was set as 40 healthy subjects and 60 acne subjects, which enables the assessment of the differences in biomarkers between healthy individuals and acne subjects, including a respective 95% confidence interval, with sufficient precision according to the size of the metabolite kit, with expert opinions in metabolomics, molecular biology, dermatology, and statistics. The gender ratio in each group was 1:1.

### Subjects

The inclusion criteria for acne group were as follows: (1) age 19–35 years, (2) body mass index (BMI) 18.5 to 23 kg/m^2^, and (3) voluntary participation in this clinical trial. The inclusion criteria for the control group were as follows: (1) age 19–35 years, (2) BMI 18.5 to 23 kg/m^2^, (3) no evidence of acne, (4) (only for female subjects) regular menstrual cycle during the last 3 months, (5) no digestive-related uncomfortable symptoms in usual situations, (6) no abdominal muscle tension, (7) other disorders that may affect the outcomes, and (8) voluntary participation in this clinical trial. The exclusion criteria for both groups were: (1) use of oral steroids, oral contraceptives, oral vitamin A derivatives, antibiotics, or herbal medicines that may affect the test results during the last 4 weeks, (2) no fasting for more than 8 hours after 9:00 pm on the day before the visit, (3) consumption of alcohol in the preceding week, (4) smoking, (5) pregnancy or lactation, (6) previously diagnosed with polycystic ovary syndrome (PCOS), (7) judgment that physical or mental testing is not appropriate for the clinical trial, and (8) participation in other clinical trials that might affect this trial.

### Outcome

#### Metabolomic profiles with pro-inflammatory cytokines

We evaluated two androgens (dehydroepiandrosterone sulfate [DHEA-S] and testosterone), serum lipids, six essential amino acids (EAAs), seven nonessential amino acids (NEAAs), three other amino acids (AAs), and eight pro-inflammatory cytokines in all subjects. More information about profiling methods are presented in a previously published protocol study [19]. Subjects in each group were divided into two groups: adolescent acne (age ≤25 years) and adult acne (age >25 years) [21].

#### PI

The assessor fulfilled the inventory for PI of acne, which is based on weighted scoring system (see S1 Table) after diagnosis of each subject [18]. After completing the questionnaire, subjects were classified into Wind-Heat (WH), Dampness-Heat (DH), Phlegm-Stasis (PS), and Disharmony of the thoroughfare and conception vessels (DTCV) pattern based on body and acne conditions (Table 2). The DTCV acne group included only female subjects, since the diagnosis is related to the menstrual cycle. Therefore, values of the DTCV acne group were compared with those of female subjects in the control group. Values of female subjects in the other PI groups were also compared with those of female subjects in the control group, to assess whether the characteristics of the female subjects in the DTCV group differed from other female subjects with acne.

**Table 2:** Characteristics of each PI.

Wind-Heat	Acne type mainly with papules that are reddish, painful with itching, have burning sensations, and sometimes have pustules.
Dampness-Heat	Acne type mainly with pustules that are easily swelling, red, painful with greasy skin, and sometimes have nodules.
Phlegm-Stasis	Acne type mainly with nodules or cysts that are hard and painful, dark in color, can be long lasting, recur easily, and remain as scars, pimples, and pigmentation.
Disharmony of the thoroughfare and conception vessels	Acne type that is closely related with the menstrual cycle, is especially exacerbated before menstruation, and tends to occur in the form of small papules located under the chin or around the mouth.

#### Disease severity

Acne severity was defined in accordance with the Korean Acne Grading System. The grade ranged from 1 to 6, where 1 = ≤ 10 papules, 2 = 11–30 papules, 3 = ≥ 31 papules; ≤ 10 nodules, 4 = 11–20 ± mild ongoing scars, 5 = 21–30 nodules ± moderate ongoing scars, and 6 = ≥ 31 nodules ± severe ongoing scars ± sinus tract (papule; acne < 5 mm, nodule; acne > 5 mm) [22]. Subjects were divided into two groups: mild acne (grade 1–3) and severe acne (grade 4–6)

#### Skin assessments

Skin assessments for skin temperature, skin stiffness (cutaneous resonance running time), and sebum levels were performed on the both facial cheeks using a model ST500 skin thermometer, model RVM600 reviscometer, and model SM815 sebumeter SM815 (all from Courage + Khazaka Electronic GmbH, Kern, Germany), respectively [23, 24]. The assessments were performed three times and average values were used. The assessments were performed under strictly controlled conditions with a room temperature of 22 ± 2°C and a relative humidity of 50 ± 10% and after the resting period of approximately 30 min.

### Statistical analyses

All statistical analyses were performed using SPSS 21 software (IBM Inc., Armonk, NY, USA). Data are presented as means ± standard deviations for continuous data or number (n) for categorical data. The baseline characteristics were compared by an independent t test for continuous values and chi-square test for categorical values. An independent t test was performed for the comparison of continuous data between the healthy subjects and acne subjects and subanalyses according to gender, age, and PI. Analysis of covariance was performed for the comparison according to severity (controls vs. mild vs. severe), after adjustment for gender. Tukey's HSD post-hoc test was used to determine which groups differed from each other. In all tests, a *p*-value < 0.05 was considered statistically significant.

## Results

### Subjects

From May 2017 to November 2017, a total of 40 healthy volunteers and 60 of acne subjects were included in this study. There were no significant differences in BMI, gender, smoking, alcohol consumption, sleep time, stress level, dietary habits, and skin assessment between two groups. A difference was evident for age (Table 3).

**Table 3:** Demographic characteristics of subjects.

Characteristics	Control (n = 40)	Acne (n = 60)	*p*
Age (years)	27.1±3.8	24.9±4.4	0.012
Body mass index	21.4±1.6	21.3±1.5	0.715
Gender (male/female)			
Total	40 (20/20)	60 (30/30)	
Adolescent (≤25 years)	12 (5/7)	35 (19/16)	0.517
Adult (>25 years)	28 (15/13)	25 (11/14)	0.586
Smoking (n)	0	5	0.061
Sleep time (<6 h / ≥6 h)	13/27	17/43	0.656
Stress response inventory	18.8±16.0	22.6±19.0	0.300
Dietary habits* (n/n, %)			
Milk	17/23 (42.5)	18/42 (30.0)	0.199
Cheese and yogurt	4/36 (11.1)	12/48 (20.0)	0.181
Bread and pasta	18/22 (45.0)	25/35 (41.7)	0.742
Cake and snacks	14/26 (35.0)	22/38 (36.7)	0.865
Fruits and vegetables	31/9 (77.5)	46/14 (76.7)	0.923
Meats	31/9 (77.5)	48/12 (80.0)	0.764
Ham and processed meat	13/27 (32.5)	23/37 (38.3)	0.552
Fish	7/33 (17.5)	18/42 (30.0)	0.157
Skin temperature (°C)	30.1±1.3	30.7±1.4	0.173
Skin stiffness (Cutaneous resonance running time) (a.u.)	109.1±39.7	101.7±	0.101
Skin sebum (μg/cm^2^)	19.2±13.8	19.2±13.8	0.785
Korean Acne Grade Scale (grade 1–3 / 4–6)	0/0	35/25	

* <3 times a week / ≥3 times a week (% of <3 times a week)

### Comparison of metabolomic profiles with pro-inflammatory cytokines between groups, and gender and age subgroup analysis

#### Overall

**Fig 1:**
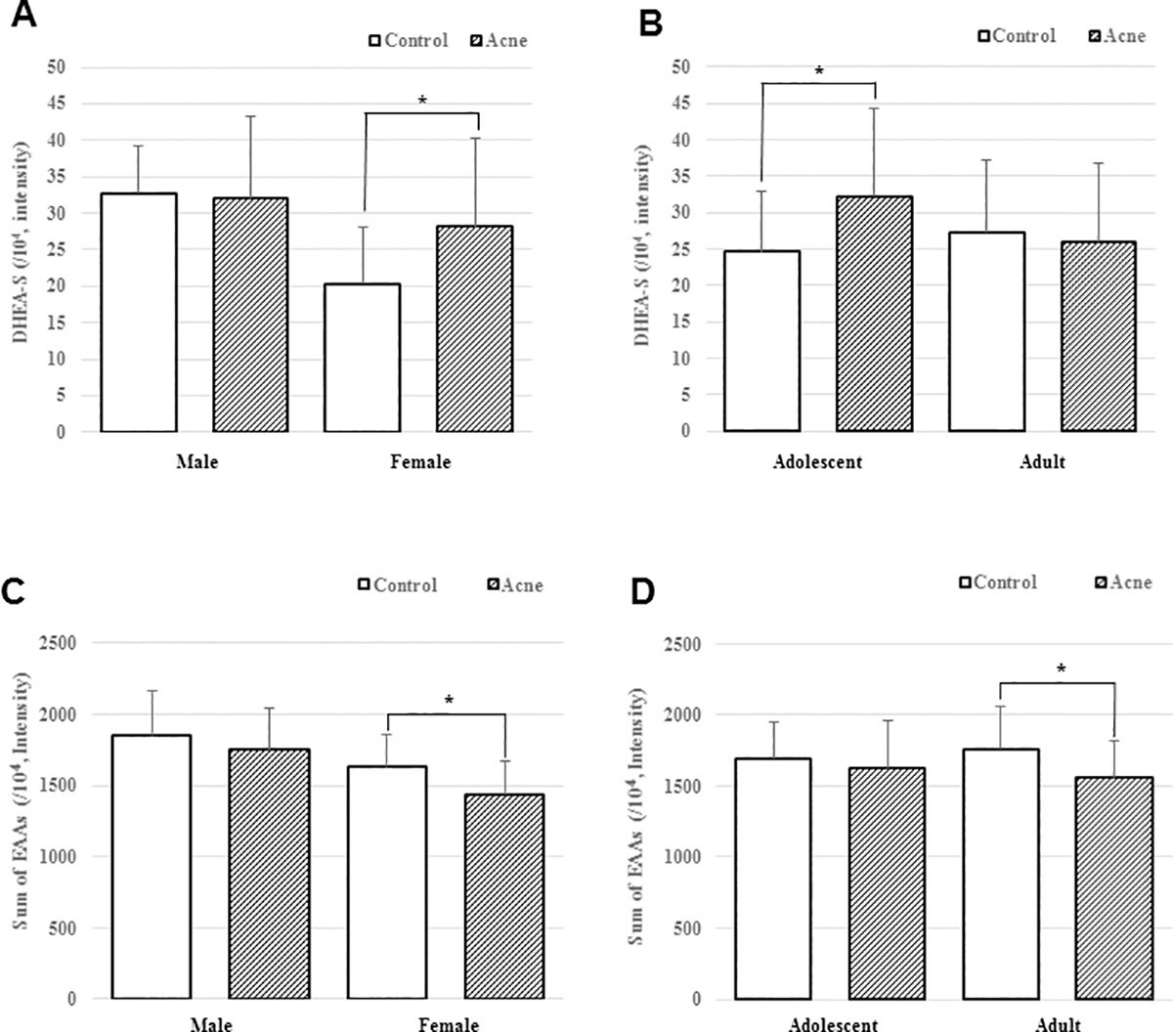
Intensity of DHEA-S and sum of EAAs in control and acne groups. (A, C) Male subjects in control group vs. acne group and female subjects in control vs. acne group. (B, C) Adolescents in control vs. acne group, and adults in control vs. acne group.

No significant differences were observed in the androgens and cytokines. Serum lipids and linoleic acids were significantly higher in the acne group. In the acne group three EAAs, two NEAAs, sum of EAAs, and sum of NEAAs were significantly lower, while two NEAAs and one of the other AAs were significantly higher (Table 4 and Fig 1).

**Table 4:** Comparison of metabolomic profiles with pro-inflammatory cytokines between groups.

	Overall			Male			Female			Adolescent			Adult		
	Control (n = 40)	Acne (n = 60)	*p*	Control (n = 20)	Acne (n = 30)	*p*	Control (n = 20)	Acne (n = 30)	*p*	Control (n = 12)	Acne (n = 35)	*p*	Control (n = 18)	Acne (n = 25)	*p*
**Androgens**															
DHEA-S^a^	26.5±9.4	29.9±11.8	0.134	32.7±6.5	32.1±11.1	0.530	20.3±7.7	28.1±12.2*	0.015	24.6±8.3	32.3±11.9*	0.044	27.3±9.9	26.0±10.8	0.660
Testosterone	1036.4±872.8	1083.5±1063.5	0.819	1743.2±690.1	2033.9±897.6	0.323	329.7±189.2	291.4±107.5	0.366	967.6±976.0	1233.7±1095.7	0.462	1065.9±842.2	840.3±986.2	0.393
**Serum lipids**^a^															
Cholesterol	5.88±1.33	5.56±2.07	0.396	5.70±1.46	5.59±2.35	0.534	6.06±1.20	5.54±1.85	0.104	6.07±1.35	5.57±2.16	0.452	5.80±1.34	5.56±1.99	0.613
Linoleic acid	1.78±1.43	2.45±1.50*	0.031	1.57±1.31	2.04±1.25	0.151	1.99±1.55	2.79±1.62	0.073	1.20±0.78	2.56±1.46*	0.004	2.03±1.58	2.27±1.58	0.602
Oleic acid	4.90±4.69	6.13±4.54	0.200	3.48±2.69	5.66±4.60	0.051	6.32±5.80	6.52±4.53	0.549	3.09±2.38	6.67±4.88*	0.019	5.67±5.23	5.26±3.89	0.762
Palmitic acid	26.3±9.8	29.0±12.4	0.244	32.7±6.5	30.9±12.2	0.556	19.9±8.2*	27.5±12.5*	0.020	24.6±8.3	30.9±13.1	0.126	27.0±10.4	26.0±10.8	0.748
**EAAs**^a^															
Isoleucine	297.4±82.5	247.0±86.5*	0.004	311.9±90.0	291.7±82.2	0.417	282.9±73.8	202.3±65.7**	<0.001	293.7±63.1	251.0±88.5	0.131	299.0±90.6	241.3±85.0*	0.021
Leucine	871.3±156.9	824.5±160.0	0.151	938.4±160.0	900.7±159.2	0.417	804.3±124.2	748.2±121.3	0.119	844.4±149.2	844.1±183.5	0.996	882.9±161.3	796.9±117.9*	0.033
Lysine	24.2±6.4	24.0±6.4	0.906	25.7±6.6	26.7±6.8	0.598	22.7±5.9	21.3±4.6	0.374	23.0±5.9	22.9±5.3	0.957	24.7±6.6	25.5±7.5	0.657
Phenylalanine	404.0±68.3	365.4±59.3*	0.002	422.5±86.5	384.9±57.1	0.070	385.5±36.9	345.9±55.8*	0.008	393.6±47.8	367.3±62.3	0.190	408.4±75.7	362.6±56.0*	0.016
Tryptophan	1.68±0.37	1.47±0.38*	0.005	1.74±0.40	1.58±0.40	0.166	1.61±0.33	1.37±0.34*	0.017	1.52±0.30	1.52±0.37	0.971	1.74±0.38	1.41±0.40*	0.003
Valine	141.8±22.6	134.0±26.1	0.090	151.5±23.3	146.3±26.9	0.483	132.2±17.4	121.6±18.8	0.051	138.8±22.2	137.1±28.0	0.846	143.1±23.0	129.6±23.1*	0.037
Sum of EAAs	1740.5±289.1	1596.3±305.1	0.020	1851.7±308.6	1751.9±295.9	0.256	1629.2±224.1	1440.7±227.3*	0.006	1695.2±255.0	1624.1±336.4	0.508	1759.8±304.8	1557.4±256.6*	0.012
**NEAAs**^a^															
Alanine	7.28±1.90	8.38±2.60*	0.025	7.55±2.14	8.15±2.31	0.357	7.02±1.63	8.61±2.89*	0.029	7.63±1.86	8.34±2.68	0.401	7.13±1.92	8.44±2.54*	0.038
Aspartic acid	0.45±0.08	0.40±0.10*	0.002	0.48±0.08	0.40±0.10*	0.010	0.43±0.08	0.39±0.09	0.093	0.46±0.07	0.40±0.10*	0.043	0.45±0.09	0.40±0.09*	0.029
Glutamic acid	2.81±1.22	3.22±1.56	0.179	3.13±1.14	3.45±1.49	0.412	2.49±1.25	2.99±1.62	0.248	2.88±1.28	3.27±1.61	0.448	2.78±1.22	3.15±1.52	0.325
Glutamine	7.01±1.78	6.50±2.64	0.057	7.12±2.02	6.63±2.73	0.495	6.90±1.56	6.37±2.59	0.418	6.29±0.98	6.66±2.60	0.629	7.32±1.97	6.27±2.73	0.112
Glycine	7.99±11.54	4.96±5.36	0.357	9.38±13.47	5.53±6.55	0.309	6.59±9.36	4.40±3.88	0.257	8.88±11.05	4.71±5.73	0.099	7.60±11.9	5.32±4.90	0.376
Serine	2.31±0.61	2.65±0.66*	0.005	2.23±0.53	2.51±0.53	0.071	2.38±0.69	2.79±0.76	0.063	2.44±0.74	2.60±0.54	0.441	2.25±0.55	2.72±0.82*	0.016
Tyrosine	83.7±17.9	74.1±12.4*	0.002	87.2±21.7	75.1±12.2*	0.016	80.3±12.8	73.1±12.7	0.056	80.2±12.9	73.9±12.8	0.149	85.3±19.7	74.5±12.0*	0.021
Sum of NEAAs	111.6±22.7	100.2±16.6*	0.005	117.0±26.2	101.8±17.2*	0.016	106.1±17.7	98.7±16.2	0.132	108.8±21.0	99.9±17.0	0.147	112.8±23.7	100.8±16.4*	0.039
**Other AAs**^**a**^															
Betaine	68.2±14.4	72.0±14.8	0.209	68.8±13.5	73.7±12.4	0.197	67.6±15.7	70.3±16.9	0.570	69.8±16.0	72.2±14.4	0.640	67.5±14.0	71.7±15.7	0.304
Hydroxyproline	2.26±0.79	2.82±1.37*	0.038	2.49±0.87	2.90±1.39	0.380	2.03±0.65	2.74±1.36*	0.036	2.39±0.83	2.92±1.47	0.249	2.21±0.78	2.68±1.22	0.093
Sarcosine	17.1±4.5	19.3±7.0	0.089	17.3±4.9	18.2±5.81	0.569	16.9±4.2	20.5±7.9	0.064	18.0±4.4	19.4±6.3	0.476	16.7±4.6	19.3±7.9	0.139
**Cytokines**															
IL-1α	5.09±2.48	5.29±3.52	0.751	5.22±2.23	5.37±4.07	0.881	4.96±2.77	5.22±2.94	0.756	5.95±2.54	5.78±3.88	0.886	4.72±2.41	4.61±2.88	0.882
IL-1β	2.23±2.65	5.97±24.36	0.343	2.52±3.22	9.32±34.67	0.388	1.92±1.92	2.73±2.10	0.180	2.19±1.26	7.81±31.42	0.542	2.25±3.10	3.29±4.92	0.367
IL-6	1.39±1.00	2.58±4.97	0.138	1.22±0.80	2.83±5.61	0.212	1.56±1.16	2.34±4.32	0.435	1.13±0.63	3.06±6.30	0.300	1.50±1.11	1.92±1.96	0.336
IL-8	35.0±71.6	40.7±100.3	0.757	20.2±31.8	53.9±134.2	0.277	49.7±95.2	27.4±45.7	0.273	29.2±42.9	35.5±58.4	0.734	37.4±81.5	47.9±140.8	0.739
IL-12β	251.5±323.0	216.6±311.3	0.590	232.4±252.0	232.2±311.4	0.997	270.5±387.1	201.1±315.6	0.490	301.4±337.5	265.7±335.0	0.752	230.0±320.4	147.8±266.0	0.318
IL-15	7.88±5.50	6.78±6.75	0.394	8.06±5.06	7.97±8.77	0.966	7.70±6.04	5.63±3.76	0.141	8.16±7.02	7.74±8.12	0.874	7.77±4.86	5.39±3.73	0.057
TNF-α	7.99±10.37	8.06±11.54	0.973	9.50±12.78	8.76±12.19	0.838	6.48±7.27	7.37±11.03	0.752	7.31±8.25	9.12±13.05	0.655	8.28±11.29	6.58±9.07	0.552
GM-CSF	12.3±8.0	11.5±25.2	0.881	11.0±14.5	12.9±30.8	0.806	13.8±21.9	10.1±18.6	0.560	16.8±25.3	13.7±30.2	0.781	10.6±14.6	8.4±15.9	0.625

Data of serum metabolites are presented as the peak area (mean ± standard deviation).

^a^Intensity was divided by 10^4^

*p<0.05

**p<0.001, compared with controls

EAA, essential amino acid; NEAA, nonessential amino acid; IL, interleukin; TNF-α, tumor necrosis factor-alpha; GM-CSF, granulocyte-macrophage colony-stimulating factor

#### Male subgroup

No significant differences were noted in androgens, serum lipids, EAAs, and cytokines. Two NEAAs and sum of NEAAs were significantly lower in the acne group (Table 4 and Fig 1).

#### Female subgroup

No significant differences were found in the cytokines. Among androgens, DHEA-S, and among serum lipids, palmitic acid were significantly higher in the acne group. Three EAAs and sum of EAAs were significantly lower, while one NEAA and one of the other AAs were significantly higher in the acne group (Table 4 and Fig 1).

#### Adolescent subgroup

No significant differences were observed in EAAs and cytokines. Among androgens, DHEA-S, and among serum lipids, linoleic and oleic acids, were significantly higher in the acne group. One NEAA was significantly lower in the acne group (Table 4 and Fig 1).

#### Adult subgroup

No significant differences were found in androgens, serum lipids, and cytokines. Five EAAs, two NEAA, sum of EAAs, and sum of NEAAs significantly lower, while two NEAAs were significantly higher in the acne group (Table 4 and Fig 1).

### Comparison of metabolomic profiles with pro-inflammatory cytokines according to disease severity

**Fig 2:**
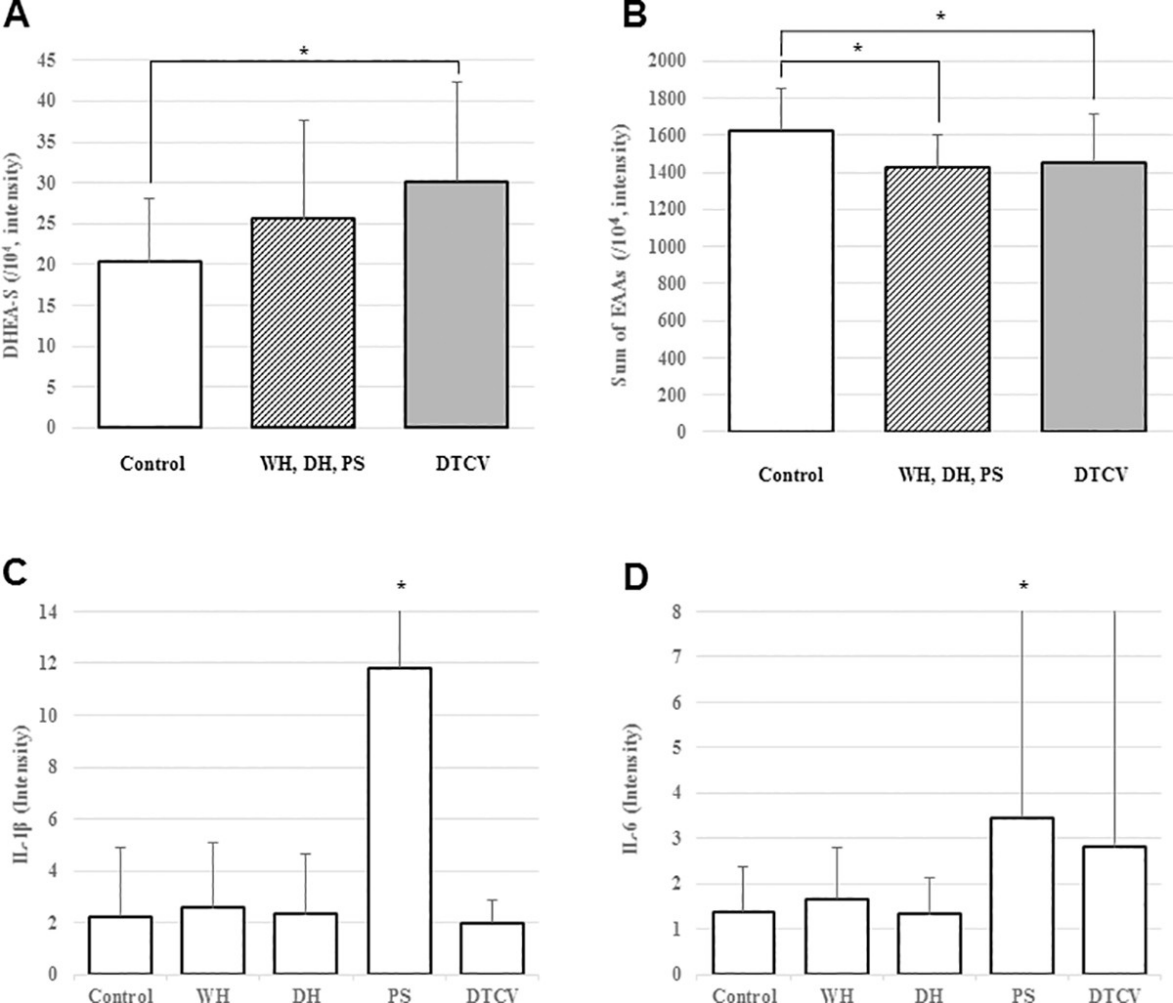
Intensity of DHEA-S, sum of EAAs, IL-1β, and IL-6 in control and PI groups. **(**A, B) Female subjects in the control group vs. female subjects in the WH, DH, and PS group vs. female subjects in the DTCV group. (C, D) Comparison between the control group and other PI groups.

No significant differences were found in androgens and cytokines. Among serum lipids, linoleic acids were significantly higher in the mild group than in the control group. One EAA and one NEAA were significantly lower in the mild group than in the control group. One EAA, one NEAA, sum of EAAs, and sum of NEAAs were significantly lower, while two NEAAs and one of the other AAs, were significantly higher in the severe group than in the control group (Table 5 and Fig 2).

**Table 5:** Comparison of metabolomic profiles with pro-inflammatory cytokines according to disease severity and PI.

	Control (n = 40)	Acne (n = 60) ^a^		PI, except DTCV^a^			Comparison between females in other groups		
		Mild (n = 35)	Severe (n = 25)	WH (n = 8)	DH (n = 13)	PS (n = 23)	Control (n = 20)	WH, DH, and PS(n = 15)	DTCV (n = 16)
**Age**	27.1±3.8	25.0±4.2	24.8±4.7	25.8±2.4	24.6±5.5	24.4±4.3	27.4±4.7	26.1±4.7	25.4±4.4
**Gender**^**b**^	20/20	19/16	11/14	4/4	10/3	15/8	0/20	0/15	0/16
**Mild/Severe**	-	-	-	5/3	9/4	8/15^c^	0/0	4/11	13/3^c^
**Androgens**									
DHEA-S^**d**^	26.5±9.4	30.4±11.6	29.2±11.6	28.6±10.8	32.1±11.1	32.4±14.1	20.3±7.7	25.6±12.1	30.2±12.2*
Testosterone	1036.5±872.8	1215.2±1113.5	885.9±975.2	1244.7±1110.5	1309±889.9	1528.1±1276.2	329.7±189.2	275.2±112.6	305.7±104.3
**Serum lipids**^**d**^									
Cholesterol	5.88±1.33	5.78±2.09	5.23±2.06	5.94±3.10	5.98±2.41	4.82±1.51*	6.06±1.20	5.09±1.96	5.94±1.71
Linoleic acid	1.78±1.43	2.65±1.62*	2.15±1.29	2.70±1.80	1.80±1.17	2.34±1.26	1.99±1.55	2.62±1.51	2.94±1.75
Oleic acid	4.90±4.69	6.86±5.08	5.04±3.43	7.04±6.24	3.99±2.68	5.96±4.06	6.32±5.80	5.43±3.82	7.49±5.00
Palmitic acid	26.3±9.8	28.9±12.7	29.2±12.3	28.6±10.8	26.3±7.1	30.8±15.3	19.9±8.2	25.6±12.2	29.2±13.0*
**EAAs**^**d**^									
Isoleucine	297.4±82.5	256.9±76.8	233.1±98.4*	258.3±57.4	252.9±77.2*	261.0±103.7	282.9±73.8	186.1±47.4**	216.4±77.0*
Leucine	871.3±156.9	826.8±138.7	821.2±188.9	880.4±121.6	848.4±126.8	848.8±187.3*	804.3±124.2	755.2190.5±102.9	742.1±138.5
Lysine	24.2±6.4	23.8±5.8	24.3±7.3	21.6±4.2	24.0±4.9	26.9±7.5*	22.7±5.9	21.6±4.2	21.1±5.1
Phenylalanine	404.0±68.3	362.3±43.2*	369.7±77.3	392.4±40.2	357.8±39.1*	368.6±69.5*	385.5±36.9	337.3±44.5*	353.4±64.6
Tryptophan	1.68±0.37	1.49±.039	1.45±0.38	1.59±0.28	1.37±0.21*	1.62±0.47	1.61±0.33	1.46±0.36	1.29±0.32*
Valine	141.8±22.6	136.4±26.2	130.5±26.2	144.4±21.0	135.3±20.5	138.5±30.7	132.2±17.4	122.2±14.5	121.2±22.3
Sum of EAAs	1740.5±289.1	1607.7±249.0	1580.3±374.8*	1656.1±231.8	1619.7±289.1	1645.4±375.4	1629.2±224.1	1423.8±184.9*	1455.4±264.1*
**NEAAs**^**d**^									
Alanine	7.28±1.90	7.83±2.41	9.15±2.72*	9.51±3.42*	7.64±2.42	8.57±2.56*	7.02±1.63	9.15±3.39*	8.15±2.37
Aspartic acid	0.45±0.08	0.41±0.08	0.38±0.11*	0.43±0.09	0.42±0.08	0.37±0.11*	0.43±0.08	0.39±0.10	0.39±0.08
Glutamic acid	2.81±1.22	3.15±1.48	3.32±1.69	3.15±0.98	2.40±0.94	3.85±1.91*	2.49±1.25	2.96±1.92	3.01±1.36
Glutamine	7.01±1.78	6.65±2.29	6.29±3.10	7.38±2.34	7.50±3.22	5.97±2.38*	6.90±1.56	6.79±2.71	6.00±2.51
Glycine	7.99±11.54	5.20±6.62	4.64±2.92	4.33±3.67	6.02±7.00	4.85±5.38	6.59±9.36	4.19±2.39	4.58±4.90
Serine	2.31±0.61	2.61±0.78	2.70±0.47*	2.56±0.70	2.36±0.63	2.75±0.42*	2.38±0.69	2.78±0.59	2.79±0.90
Tyrosine	83.7±17.9	75.4±11.9*	72.4±13.2	82.4±13.8	73.5±10.5*	72.2±12.0*	80.3±12.8	73.0±12.8	73.2±13.0
Sum of NEAAs	111.6±22.7	101.2±16.3	98.9±17.3*	107.6±20.5	99.9±14.7	98.6±17.3*	106.1±17.7	99.3±17.2	98.2±15.8
**Other AAs**^**d**^									
Betaine	68.2±14.4	72.8±15.1	70.8±14.6	62.7±12.2	73.2±15.5	73.6±13.4	67.6±15.7	66.8±16.9	73.3±16.9
Hydroxyproline	2.26±0.79	2.79±1.31	2.86±1.47	3.15±1.53	2.55±1.07	2.90±1.57	2.03±0.65	2.72±1.51	2.76±1.26*
Sarcosine	17.1±4.5	17.9±6.9	21.4±6.7*	23.0±11.6*	17.1±5.9	20.1±6.07*	16.9±4.2	22.9±9.4*	18.4±5.7
**Cytokines**									
IL-1α	5.09±2.48	5.08±3.64	5.58±3.40	5.77±4.49	5.2±2.82	6.05±4.47	4.96±2.77	6.6±3.8	4.05±1.04
IL-1β	2.23±2.65	8.04±31.60	2.95±2.15	2.58±2.48	2.34±2.32	11.83±38.74*	1.92±1.92	3.6±2.7*	1.98±0.91
IL-6	1.39±1.00	3.03±6.45	1.95±1.05	1.66±1.13	1.33±0.81	3.46±6.31**	1.56±1.16	1.8±1.0	2.80±5.90
IL-8	35.0±71.6	46.10±123.45	33.03±55.19	31.9±47.85	10.44±7.41	75.2±152.62	49.7±95.2	35.9±56.1	19.91±34.42
IL-12β	251.5±323.0	197.30±301.65	243.63±328.57	383.37±471.98	229.49±291.76	244.23±339.61	270.5±387.1	335.9±424.3	83.03±68.32
IL-15	7.88±5.50	6.84±7.61	6.70±5.53	6.72±5.04	7.37±3.88	8.38±9.77	7.70±6.04	**7.5**±4.8	4.03±1.32*
TNF-α	7.99±10.37	7.14±11.22	9.36±12.10	11.69±14.94	6.64±5.91	10.98±15.28	6.48±7.27	12.1±14.9	3.21±1.69
GM-CSF	12.3±8.0	11.22±29.92	11.95±17.19	15.46±25.37	9.77±12.89	16.98±36.33	13.8±21.9	18.1±25.1	3.12±1.15

Data of serum metabolites are presented as the peak area (mean ± standard deviation)

^a^*p*-values were calculated after adjustment for gender

^b^male/female

^c^difference in mild/severe proportion

^d^intensity divided by 10^4^

*p<0.05

**p<0.001, compared with controls

EAA, essential amino acid; NEAA, nonessential amino acid; IL, interleukin; TNF-α, tumor necrosis factor-alpha; GM-CSF, granulocyte-macrophage colony-stimulating factor

### Comparison of serum metabolites and pro-inflammatory cytokines according to PI

#### Wind-heat

No significant differences were noted in androgens, serum lipids, and cytokines. One NEAA and one of the other AAs were significantly higher in the WH acne group than in the control group (Table 5 and Fig 2).

#### Dampness-heat

No significant differences were noted in androgens, serum lipids, and cytokines. Three EAAs and one NEAA were significantly lower in the DH acne group than in the control group (Table 5 and Fig 2).

#### Phlegm-stasis

Subjects with severe acne symptoms displayed no significant differences in androgens. Among serum lipids, cholesterol was significantly lower in the PS acne group than in the control group. Two EAAs, three NEAA, and sum of NEAAs were significantly lower, while one EAA, three NEAAs, and one of the other AAs were significantly higher in the PS acne group than in the control group. Among cytokines, interleukin (IL)-1β and IL-6 were significantly higher in the PS acne group than in the control group (Table 5 and Fig 2).

### Disharmony of the thoroughfare and conception vessels

Among androgens, DHEA-S was significantly higher in the DTCV acne group than in the female subjects of the control group, while no significant differences were observed among the female subjects in other PI groups. Among serum lipids, palmitic acid was significantly higher in the DTCV acne group than in the female subjects of the control group, while no significant differences were observed among the female subjects of other PI groups. Among EAAs, isoleucine, tryptophan, and sum of EAAs were significantly lower in the DTCV acne group than in the female subjects of the control group. Isoleucine, phenylalanine, and sum of EAAs were significantly lower in the female subjects in other PI groups than in those of the control group. Among NEAAs, sarcosine was significantly higher in the female subjects of other PI groups and hydroxyproline was significantly higher in the DTCV acne group than in the female subjects of the control group. Among cytokines, IL-15 was decreased in the DTCV acne group, while IL-1β was increased in the female subjects in the other PI groups compared with the control group (Table 5 and Fig 2).

## Discussion

Acne vulgaris has been suggested to be closely related with mammalian target of rapamycin complex 1 (mTORC-1) driven metabolic diseases [25], which include obesity [26], type 2 diabetes mellitus [2], and cancer [27]. Western diet, which is characterized by the high intake of hyperglycemic carbohydrates and dairy products, increases insulin and insulin-like growth factor-1 (IGF-1) [28, 29], and promotes mTORC-1 signaling, the key regulator of anabolism and lipogenesis [30, 31]. mTORC-1 activation induces hyperseborrhea and hyperkeratinization of the pilosebaceous duct by stimulating androgens, and dysseborrhea by changing the proportion of free fatty acids (FFAs) in sebum. IGF-1 induces pro-inflammatory cytokine expression in sebocytes by activating the nuclear factor-kappa B pathway [25].

In this study, DHEA-S was elevated only in female subjects and adolescents with acne. DHEA-S was not elevated in the overall subjects, male subjects, and adults with acne. Previous studies reported that the rise in DHEA-S correlates with the onset of acne in children, and higher levels of DHEA-S are found at puberty in girls with acne than in those without acne [32]. Subjects with the highest levels of DHEA-S develop the most severe acne among adolescent girls [33]. Hormonal factors are also important in adult acne. It was reported that 62% of adult women with acne experience worsening of their acne in the days before menstruation [34]. However, the majority of adult acne subjects have normal androgen levels in the absence of PCOS, congenital adrenal hyperplasia, and adrenal or ovarian tumors [35]. This suggests that initiation of acne symptoms is induced with elevated androgens in puberty, especially in girls, and with the persistent progression of acne into adulthood; hormonal receptors expressed by both sebocytes and keratinocytes may be more sensitive to low levels of androgens [35, 36]. Meanwhile, only female subjects in the DTCV group displayed elevated DHEA-S compared with those in the control group, despite the larger proportion of subjects with mild symptoms. The DTCV pattern displayed a weighted value for the effect of the menstrual cycle concerning the worsening of acne, menstrual irregularity, and pain in the lower abdomen (See S1 Table). Among these symptoms, menstrual irregularity is the most representative symptom of PCOS. Although we excluded subjects with previously diagnosed PCOS and obese subjects during enrollment, subjects with undiagnosed PCOS may not have been fully excluded, since PCOS is a common but often undiagnosed condition [37]. Therefore, many female patients with undiagnosed PCOS visit dermatological clinics to treat their acne. Dietary correction and hormonal therapy are more helpful for patients with PCOS. However, hormonal testing is not frequently performed due to its cost and inconvenience of blood collection. Therefore, determining the PI pattern after the completion of a single questionnaire may improve the clinical efficiency of treatment for female patients.

Several studies have reported quantitative changes of sebum (hyperseborrea) and qualitative modifications of sebum (dysseborrhea) with higher amounts of FFA in skin affected by acne [38–40]. Elevated IGF-1 and insulin that is mediated by western diet increases sebaceous gland growth and sebaceous lipogenesis, which causes hyper- and dysseborrhea in acne patients [25]. Concerning serum lipids, acne patients frequently display abnormal lipid profiles, including total cholesterol, triglyceride, low density lipoprotein cholesterol, and lipoprotein [41, 42]. The present study is the first to evaluate serum FFAs in patients with acne. The data are consistent with the observation of elevated FFAs in insulin resistant subjects [43], and the increases of linoleic acids (polyunsaturated FFA), oleic acids (monounsaturated FFA), and palmitic acids (saturated FFA) in acne subjects. These findings indicate that serum FFAs as well as sebum FFAs are elevated in patients with acne. Consistent with results for DHEA-S, FFAs were elevated only in female subjects and adolescents, not in the male subjects and adults. Androgens stimulate lipid synthesis [44], and the upregulation of androgens and serum lipids affects acne in female subjects and adolescents. Additional lipidomics data are needed to clarify the lipid mechanism in acne subjects.

No studies have reported the profile of serum AAs in acne. In this study, sum of EAAs and NEAAs were decreased in the overall, female, adult, severe acne, and PS acne subgroups. The PS group had weighed value for inflammatory lesion, pustule, cyst, nodule, and pigmentation (see S1 Table). Consistent with our observations, decreases in serum AAs have been described for several chronic inflammatory disorders, including rheumatoid arthritis, inflammatory bowel disease, and chronic kidney disease [45–47]. Concerning dermatology, AAs chelate calcium ions and stimulate exfoliation of the stratum corneum, accelerating the turnover of the stratum corneum [48]. Therefore, decreased serum levels of AAs may induce hyperkeratinization, one of the four major factors of acne. However, topical exfoliating therapies can have a negative impact on the barrier functions by increasing transepidermal water loss [49]. Our findings indicate that AAs may be efficiently used to improve skin affected by acne, especially in adults, females, and those with severe acne.

IGF-1 and *Cutibacterium acnes* are the most important factors that induce an inflammatory response in acne. A recent study of biopsy samples from acne subjects showed that expression of pro-inflammatory cytokines is upregulated after stimulation of IGF-1 [50, 51]. *C*. *acnes* induces production of local pro-inflammatory cytokines, including IL-1β, IL-8, IL-12 and tumor necrosis factor-alpha (TNF-α) via a Toll-like receptor 2 dependent mechanism [52, 53]. The present study is the first to evaluate the serum levels of IL-1α, IL-1β, IL-6, IL-8, IL-12β, IL-15, TNF-α, and granulocyte-macrophage colony-stimulating factor in acne subjects compared with the levels in healthy controls. No significant differences were observed in the acne group and all subgroups, including the severe acne subgroup. However, the PS group, which had weighted values for inflammatory lesions, pustules, cysts, nodules, and pigmentation (see S1 Table), exhibited elevated serum levels of IL-1β and IL-6. This suggests that IGF-1 and *C*. *acnes* activate local inflammation in skin lesions. However, the systematic impact may not be significant, except in patients who develop many inflammatory lesions.

There are several limitations to this study. First, 60 acne subjects were divided into four PI groups. The number of subjects in each PI group may not have been large enough to obtain statistical significance. Secondly, acne subjects with undiagnosed PCOS may not have been fully excluded, and this could have biased the analysis of female subjects. Nevertheless, in the real world, blood or ultrasound tests are not frequently performed for patients. Therefore, including subjects with undiagnosed PCOS could better reflect actual clinical practice.

This is the first study to investigate serum lipids, AAs, and cytokines in subjects with acne. As an exploratory study, additional dedicated studies are needed to confirm the results. Prospective studies with more patients are needed to confirm the characteristics of each PIs, and future studies that provide more lipidomics data may clarify the lipid mechanism.

## Supporting information

S1 Table Pattern diagnosis inventory of acne. (DOC)
