# Supplementary material for: Integrated targeted serum metabolomic profile and its association with gender, age, disease severity, and pattern identification in acne
Source: PLoS One. 2020 Jan 17;15(1):e0228074. doi: 10.1371/journal.pone.0228074 (PMC6968861; doi:10.1371/journal.pone.0228074)
Supplement: S1 Table — (DOC) [file pone.0228074.s001.doc]

S1 Table. Pattern diagnosis inventory of acne

|  |  | Weighted value | | | |
| --- | --- | --- | --- | --- | --- |
|  |  | WH | DH | PS | DTCV |
| Objective | | 16.35 | 18.17 |  |  |
|  | Inflammatory lesion of acne |  |  | 14.88 |  |
|  | No inflammatory lesion of acne | 12.21 | 22.49 |  |  |
|  | Pustule |  | 13.40 | 16.44 |  |
|  | Cyst |  |  | 18.88 |  |
|  | Nodule |  |  | 17.97 |  |
|  | Pigmentation |  |  | 13.85 |  |
|  | Oily skin |  | 26.19 |  |  |
|  | Facial blushing | 14.38 |  |  |  |
| Subjective | |  |  |  |  |
|  | Itching on acne lesions | 15.87 |  |  |  |
|  | Burning signs on acne lesions | 15.73 |  |  |  |
|  | Swelling on acne lesions | 12.61 | 19.75 |  |  |
|  | Pain on acne lesions | 12.85 |  |  |  |
|  | Acne occurs around mouth and jaw |  |  |  | 17.42 |
|  | Skin symptoms repeat and continue for a long time |  |  | 17.98 |  |
|  | Menstrual cycle effect on severity of acne |  |  |  | 42.66 |
|  | Menstrual irregularity |  |  |  | 22.37 |
|  | Pain in lower abdomen |  |  |  | 17.55 |
